# A “stick to beat you with”? Advocating for a Critical Close Reading of ‘Vocation’ Among Evangelical Medics in England

**DOI:** 10.1007/s10943-022-01564-y

**Published:** 2022-05-09

**Authors:** Jennifer Riley

**Affiliations:** Bay Cottage, 1 Ropewalk, Shoreham-by-Sea, West Sussex, BN43 5WW England, UK

**Keywords:** Evangelicalism, Vocation, Identity, Healthcare, *Beruf*, UK, England

## Abstract

Evangelical Christianity and healthcare work are two contexts in which vocation is often an important discourse. Exploring uses, understandings and implications of vocation for evangelical medics thus offers a rich opportunity to critically interrogate vocation from two important perspectives. In addition to identifying a three-tiered construction of vocation, on macro-, meso- and micro-levels, this paper suggests that to fully understand its manifestations among a sample of English evangelical medics, a critical, Weberian-style reading is valuable. This latter conclusion resonates with those drawn by scholars who extend a critical view across constructions of medical vocation more broadly, not least given concerns regarding workplace burnout.

## Introduction

‘Vocation’ is a slippery term. To one, it might refer to a specific calling to a religious life, just as well as it might, to another, evoke earnest dedication to a secular career path by someone with no religious interests. Its usage is often imprecise and quite rarely interrogated. Yet in some circles, vocation remains an important discursive concept and a significant element of how individuals understand and interpret their work and identity. Healthcare and medical work are important examples, with scholarship on medical vocation often linked to contemporary concerns regarding workplace burnout (Nussbaum, 2018, p.426). Evangelical Christianity is another such context, where ‘vocation’ and ‘calling’ are often used interchangeably (Williams, 2013, p.255). Nevertheless, beyond calling to Christian ministry, these ideas have received limited empirical and theological exploration in evangelical circles, particularly in recent years.

This paper situates medical vocation within the context of evangelical Christianity, using insights gained from a recent qualitative study with evangelical medics in England. It examines the medics’ constructions of ‘calling’ in depth and the implications of these. These participants’ socialization into two contexts where vocation can be a significant discourse positions them well to offer considered, rich reflection on its nature and implications among both healthcare workers and evangelical Christians. This paper illustrates the participants’ varied language and experiences, evident within a widely shared, though nuanced, evangelical logic of vocation operating on macro-, meso- and micro-levels. This three-tiered model offers a more precise framework for understanding evangelical constructions of calling and vocation than has been offered before. Within it are contained both vocation’s positive and neutral depictions, and its ambiguous and negative ramifications, not least as the three levels of the discourse interact. I thus argue that we might beneficially draw insights from Weber’s work on vocation in order to recognize that a sense of personal calling can be ambiguous or burdensome. This observation, in turn, highlights anew the importance of recognizing this range of implications, not least so that evangelical medics for whom vocation is an important aspect of their self-understanding might be appropriately supported. Yet this observation is not unique to evangelical medics. As such, the paper echoes those who would critically examine medical vocation more broadly, suggesting that recognizing vocation’s sometimes ambiguous and negative manifestations might facilitate deeper understanding and, perhaps, greater support for evangelicals and medics.

## Literature Review

### Max Weber’s Work on Beruf

Max Weber (1864–1920) remains an authoritative starting point for discussions concerning vocation, especially where Protestant Christianity is in view. *The Protestant Ethic and the Spirit of Capitalism (Die protestantische Ethik und der Geist des Kapitalismus)* (1905) contains Weber’s most famous presentation of calling (*Beruf*), integral in his non-Marxist history of the emergence of Capitalism. We find his broader understanding, divorced from this specific historic argumentation, in the ‘Vocation Lectures’ of, respectively, 1917 and 1919: *Science as Vocation* (*Wissenschaft als Beruf)* and *Politics as Vocation* (*Politik als Beruf).* In *Wissenschaft,* Weber discusses scientific vocations in relation to contemporary German university structures and postgraduate academic prospects (Weber, 2004, p. 1). *Beruf*’s felt dimensions come across strongly in *Wissenschaft,* where Weber describes the scientist’s ‘devotion,’ ‘passion’ and ‘strange intoxication,’ divorcing these emotions from technical, utilitarian scientific outcomes (Weber, 2004, pp. 8–9, 14). In *Politik,* he poses the ‘general question of what politics is as a vocation and what it can mean’ (Weber, 2004, p. 32). Bosk applies Weber’s insights to his own academic career in medical sociology, highlighting the illuminating power of Weber’s insights some century after he first presented them (even as Bosk highlights ways in which his and others’ senses of vocation might deviate from Weber’s ‘ideal type’, not least insofar as one may ‘drift’ into one’s calling rather than following an impassioned call from an inner voice) (Bosk, 2014, pp. 376–377).

Taken together, the Vocation Lectures confirm that Weber both divorced *Beruf* from specific religious frameworks and recognized its relevance for individuals alongside its historical social significance. We might thus take an individualized framing of *Beruf* and locate it within Weber’s social argument in the *Protestant Ethic.* There Weber describes *Beruf* as ‘systematic work in a worldly calling,’ a ‘task set by God’ for an individual within ‘a definite field’ (Weber, 2013, p. 35). Weberian *Beruf*, then, involves other-worldly purpose embedded in a specific, but every-day, secular activity: as he explains, this ‘inevitably [gives] every-day worldly activity a religious significance’ (Weber, 2013, p. 36).

### Contemporary Treatments of Vocation

More recent literature on vocation, and its manifestations among medics, evangelicals and religious medics more broadly, can be helpfully grouped into three categories. Literature in the first category makes reference to vocation or calling but offers limited exploration of these concepts’ meaning. For example, Puchalski and Ferrell (2010) offer no expansion when they suggest that ‘by being attentive to one’s own spirituality and especially one’s sense of sacred call to service to others, clinicians may be able to find more meaning in their work and hence cope better’ (p. 175). This does show, however, that calling and vocation are terms which resonate in medical contexts quite apart from any particular religion and certainly beyond the realms of work within religious contexts and communities. Similarly, Chen et al. (2019) ally vocation to a ‘sense of mission and purpose,’ but do not discuss its relationship to any religious affiliation, and acknowledge that their measures of this ‘mission and purpose’ were insufficiently precise (pp. 664–669). Cadge (2013) offers some detail in her study *Paging God,* a qualitative exploration of the relationship between religion and medicine conducted in the USA, working with doctors, nurses and chaplains in intensive care units. Cadge describes ‘several physicians [who] spoke of deriving a sense of vocation from their religious upbringing,’ allying it to a sense of inevitability, being ‘where I should be,’ and destiny (pp. 168–169). She uses the term in relation to both hospital chaplains and medics, further illustrating that vocation can characterize both religious and secular work (Cadge, 2013, pp. 186–187).

The literature in the second category awards vocation more central and often more detailed investigation. From a theological perspective, Fowler’s work is a significant example. Fowler used Piaget’s work on childhood development as a springboard for a speculative ‘stage theory’ of religious faith development within the Christian tradition. While vocation is not the central focus of Fowler’s work, it features at salient points. For example, in a manner reminiscent of Weber’s ‘definite field,’ Fowler links an individual’s specific work and circumstances with God’s work in the world, describing vocation as ‘a purpose…that aligns your life with the purposes of God’ (Fowler, 1991, pp. 126). Fowler thus closely associates Christian vocation with being ‘urged into the world’ in ‘service’ (1991, p. 50).

Williams’ exploration of calling among evangelical international students studying in USA universities is a rare focussed sociological exploration of contemporary evangelical understandings of calling, and, how these understandings affect their lives. Testament to Weber’s enduring significance, Williams takes *The Protestant Ethic* as his starting point. He defines calling as an ‘interpretive lens,’ grounded in belief that God is active in the world, leading believers to see themselves and their work as religiously significant and part of God’s ‘plan’ despite its secular setting, echoing Weber’s conceptualization of a ‘task set by God’ to ‘work in a worldly calling’ (Williams, 2013).

### Positive Presentations

Fowler and Williams both further echo Weber in emphasizing vocation’s potentially positive implications. In *Politik* Weber describes one who lives ‘for’ politics, making it ‘his “life” in an *inward sense*’ and whose ‘inner equilibrium and…self-esteem’ are bolstered by ‘the consciousness that by serving a “cause” he gives his own life meaning’ (Weber, 2004, p. 40 [emphases original]). Similarly, Williams notes that a sense of calling provided the students in his study ‘strength, support and guidance’ (Williams, 2013, p. 257). This shaped the participants’ views of the future: having seen God’s facilitation of what they understood to be his purposes for their lives, they inferred confidence in God’s ongoing direction (Williams, 2013, pp. 264–267). Fowler too suggests vocation can give ‘coherence and larger purpose…integrity, zest and meaning’ and thus support and sustain (Fowler, 1991, p. 120).

Vocation’s positive consequences are also illustrated in healthcare literature. We noted above Puchalski and Ferrell’s observation that attending to a sense of calling may help clinicians ‘cope’ or find meaning (2010, p. 175). Several participants in Cadge’s study found comfort in their ‘sense of vocation…a feeling that they were called or destined by a higher power to become a physician’ (2013, p. 168). Several chaplains explained that, coupled with a sense of ‘an intimately present God,’ their sense of calling helped them avoid burnout (Cadge, 2013, p. 168). Astrow (2013) also explores the relationship between vocation and burnout, arguing that reclaiming the notion that medicine is a spiritual vocation presents one means of responding to the ‘major concern’ of widespread physician burnout in the USA. Vocation emerges as potential counterbalance to the business, financial and market considerations that many consider antithetical to the pursuit of medicine, emphasizing instead ‘powerful forces’ of ‘intrinsic motivation’. Like Astrow, Hallam (2002) also somewhat wistfully compares pre-NHS ‘quasi-religious’ conceptualizations of nursing work as a ‘duty to the profession founded in an ethic of vocational care’ to its contemporary framing as a ‘secular occupational identity’ witnessing a ‘crisis’ of recruitment and retention (pp. 35–36). Less wistfully, Jackson, Anderson and Maben (2021) point out that presenting nursing as ‘labour’ is a ‘positioning [which] challenge[s] the view of nurses as angels, whose work is vocational and altruistic, rather than as highly skilled paid workers.’

### Negative and Ambiguous Implications

The literature in the third category draws attention to vocation’s negative and ambiguous implications. This third category returns us to Weberian *Beruf.* While, as shown above, Weber was attuned to its potentially positive ramifications, his portrayals of *Beruf* are by no means purely positive or neutral. Alongside his infamously bleak view of the future amid modernity’s ‘iron cage of rationality’ at the end of *The Protestant Ethic,* Weber also notes potentially damaging effects among *Beruf’s* ‘far-reaching psychological consequences’ (Weber, 2013, p. 92). He suggests, for example, that *Beruf* can facilitate exploitation, noting that the evolution of Protestant Asceticism ultimately ‘legalized the exploitation of this specific willingness to work’ (Weber, 2013, p. 106). *The Protestant Ethic* also presents *Beruf* as a double-edged sword which, while edifying, can become an exclusive, burdensome duty, perceived as ‘[t]he only way of living acceptably to God… solely through the fulfilment of the obligations imposed upon the individual’ (Weber, 2013, p. 36). In the Vocation Lectures, Weber suggests this calling-obligation risks diminishing all else in life, as one becomes ‘wholly devoted to his subject’ and ‘live[s] only for’ it (Weber, 2004, pp. 7, 10). Similarly, in *The Protestant Ethic*, he notes that Puritans harboured suspicion and hostility towards all ‘impulsive enjoyment’ beyond one’s calling (Weber, 2013, p. 98).

Nussbaum’s, 2018 article ‘The Worthless Remains of a Physician’s Calling’ takes Weber’s *Wissenschaft* and *Protestant Ethic* as interlocutors, noting that ‘Weber understands the rationalization of the Calvinist version of calling to have enabled the development of capitalism, which then repaid the favour by thinning the vocation into worthless remains, a ghostly version of a vocation’ (p. 422). He contrasts this to Osler’s work, which has been widely cited as suggesting that a sense of vocation can bolster medics (Nussbaum, 2018, p. 426). In light of physician burnout in the USA, Nussbaum is reluctant to allow Osler’s depiction of vocation to continue oppressing by means of its unattainable positivity. Rather, he suggests that ‘[w]ith a Weberian appreciation…physicians might be more realistic about the practice of medicine’ and ‘abandon romanticized accounts’ of medical vocation (Nussbaum, 2018, p. 426).

Harrison & Innes’, 1997 ‘Medical Vocation and Generation X’ also identifies reasons for treating presentations of medical vocation with wariness. They suggest medics’ vocational commitment and resultant willingness to be self-sacrificial can lead to junior staff in particular being placed under ‘unfair pressure’ by senior staff (Harrison & Innes, 1997, pp. 11–12). Further echoing Weber’s observations regarding the ‘exploitation of… willingness to work,’ Harrison and Innes go on to propose that doctors may also put unfair pressure upon themselves, tolerating longer hours and more stressful shifts than they ought, with adverse potential consequences for themselves and their patients (1997, pp. 11–12, 23). Correspondingly, Rafferty’s *The Politics of Nursing Work* traces an uneasy relationship between nursing, vocation and exploitation to 1930s Britain, noting that ‘nurses were frequently overworked, underpaid and compelled to undertake work which ought to be done by other people. Nursing would nevertheless always be a calling apart, a service based on vocation’ (1996, pp. 149–150). Harrison and Innes also cite Volf’s concern that vocation encourages work’s ‘divinization,’ whereby it becomes valued at the expense of other aspects of life (Harrison & Innes, 1997, p. 19; Volf, 1991, pp. 107ff). This bears close similarity to Weber’s suggestion that a calling can become all-consuming.

Traynor’s work sits ambiguously here. On the one hand, in *Managerialism and Nursing* (1999) he suggests that a discourse of vocation might reconcile a presentation of ‘caring as morally worthwhile, intrinsically satisfying and even empowering’ with nurses’ struggles with ‘exploitation and disempowerment in the workplace’ manifest in, for example, their work’s encroachments on their personal time and having to ‘put themselves out’ in order to do their job well (pp. 147–148). Thus, like Rafferty and Astrow, Traynor presents vocation, and its connotations of self-sacrifice, as an antidote to the burdens contemporary medicine places upon its practitioners, and thus to exploitation. By contrast, his article ‘Autonomy and Caring’ (2019) notes that in the early twentieth century, in both the USA and Europe, medicine ‘develop[ed] cults of loyalty, with their badges, and uniforms, an ideology of vocation and belongingness that covered over exploitation’ (p. 5). Traynor hopes his article might expose such exploitation. Traynor thus both credits vocation with the potential to combat exploitative discourses, and presents it as potentially complicit in exploitation. In sum, Traynor’s work therefore illustrates the importance of exploring vocation carefully and critically.

This paper will show that negative and ambiguous facets are important dimensions of how vocation and calling are experienced among evangelical Christian medics. Those seeking to understand evangelicalism and medicine’s relationship—including those seeking to support evangelical medics—might helpfully follow Weber and his contemporary echoers, viewing *Beruf* with an eye to its potentially negative consequences. Noting concerns regarding the relationship between vocation and exploitation, guilt and the diminishment of other areas of life—not unique to evangelical medics—this paper also advances the ‘third category’ of literature, encouraging broader critical examination of medical vocation.

## Methodology

### Autobiographical Elicitation

This paper draws upon data gathered for a research project exploring the relationship between healthcare work and evangelicals. The project used an autobiographical elicitation methodology in conversation with 23 participants, all doctors or nurses with experience working and/or training in the NHS in England, all of whom self-identified as evangelical Christians. Autobiographical elicitation begins with the participant writing or dictating personal reflections, guided but not constrained by suggested topics and themes provided by the researcher. These reflections are returned and used to develop individual semi-structured interview schedules to probe for further depth and comparative insights.

The two-stage elicitation methodology offered several clear advantages over interviewing alone. First, it meant that each participant’s interview was based around themes and topics they deemed important. This took seriously the participants’ agency and mitigated the extent to which interview schedules risk being defined primarily by the researcher, rather than by the researched whose experiences are in view (Schielke & Debevec, 2012, p. 3). Secondly, since both researcher and interviewee had had a chance to consider each other’s interests beforehand, the time these often busy participants could allot to an interview was utilized maximally, avoiding irrelevant and hypothetical topics, emphasizing instead that which the participants had already signalled to be important (Davies, 2008, pp. 105–106; Hollway & Jefferson, 1997, p. 55). Thirdly, the lengthier process facilitated the development of rapport between researcher and interviewee, in turn generating honesty and openness conducive to discussing some of the sensitive topics which arose.

The initial ‘reflection’ stage saw the medics recording reflections on the relationship between their work and faith. Participants were guided—though expressly not restricted—by a list of prompts evoking themes of interest to the project, and encouraged to tell stories and provide examples. While some stuck closely to the suggestions, others used them as a springboard to describe their work, faith, and their interaction using their own headings and frameworks. Several, for example, used a chronological presentation as a starting point. I explained that participants were welcome to record reflections in any format they wished: while most chose to write their reflections, by hand or on a computer, some opted for audio-recordings (these were subsequently transcribed verbatim). I recommended taking four weeks to complete the exercise, leaving participants to decide whether to complete it in ‘one go’ or add regular entries. Participants had a range of preferences and approaches, some using the recommended four weeks, where others requested longer.

The subsequent ‘interview’ stage used these autobiographical reflections as a starting point for eliciting further insights. Each semi-structured interview schedule was designed in dialogue with the participant’s reflections. Some questions sought to elicit further insights and depth about what the participant had reported, where others addressed topics they had not raised, but were emerging as important comparative themes in other interviews and reflections. Vocation was introduced as a topic and discourse in both ways, the researcher raising it if it seemed relevant or applicable but had not been raised by the participant, with consideration for the interview’s flow and time-management. Subsequent coding located vocation, calling and related themes in all but four participants’ narratives, suggesting its widespread relevance in the study. Interviews had a mean length of 54 min.

### Recruitment and Sampling

While it is important not to make unwarranted claims regarding generalizability and representativeness, this methodology generated 250,000 words of rich, comparable data centred on individual narratives and experiences. Furthermore, I carefully monitored both ongoing recruitment and the resulting sample for balance and breadth with respect to several demographic variables. The sample intentionally captured multiple medical specialities, including doctors who had already specialized in: psychiatry/child psychiatry; neurology; surgery; Accident and Emergency (A&E); General Practice (GP); geriatrics; and palliative and hospice medicine. Since different career stages present different challenges and responsibilities, it was important to include: trainee medics; those newly qualified and yet to specialize in a particular field (juniors); those who had already specialized (specialists); and retirees. The spread of career stages also controlled for age, and changes in English medical culture, law and training since the late 1960s. I also ensured a balance of genders, and included some who had trained outside Britain, as well as individuals of black, Asian and minority ethnicity (BAME). I use pseudonyms for all participants. As such, the study incorporated a diverse range of experiences (see Table 1). Since analysis drew upon insights, themes and ideas present across this diverse sample, we can take confidence that the resulting conclusions will resonate with a certain breadth of evangelical medics working in the NHS in England.

**Table 1:** Sample Demographics at time of interview

Gender					Career stage								
Male	Female				Trainee		Junior		Specialist			Retired	
13	10				3		5		10			5	

Work pattern^a^					Ethnicity								
Full time	Part time				White British		Other						
18	5				19		4						

Region(England)													
North East	North West				Midlands		South East/London						
8	1				7		7						

Specialties^b^														
Child psychiatry/psychiatry	General Surgery				General Practice		Accident and Emergency(A&E)		Neurology			Palliative/Hospice		Geriatrics
4	1				8		1		1			2		1

^a^The work pattern that characterized the majority of their training or career up to the time of the interview

^b^Some were yet to specialize in a particular medical field; others had specialized in different fields at different times. All had experience working in a variety of fields as part of their medical training. As such, this section does not total 23

Recruitment was purposive, augmented by snowball and opportunity sampling. I contacted regional Christian Medical Fellowship (henceforth CMF) groups, whose leaders shared my call for participants. Participants then regularly offered to put me in touch with other evangelical medics known to them through their churches or professional networks. I managed both strands of recruitment in dialogue with ongoing monitoring of the sample’s demographic composition to avoid undue homogeneity, and at times requested that participants might put me in touch with people in underrepresented categories. This second recruitment avenue meant I was able to include those who described themselves evangelical yet eschewed the CMF because of its conservative public discourse, theology and ethical positions. As such, while all participants self-described as ‘evangelical’ (this self-description was the criterion for inclusion) this term held diverse meanings for participants, who variously adopted and eschewed more precise descriptors (‘conservative,’ ‘open,’ or ‘liberal). In this sense, this study reflects British evangelicalism’s diversity (Guest, 2008, p.20). While the CMF defines evangelicalism using a 10-point ‘statement of faith’ (Christian Medical Fellowship, 2021), it is more helpful to locate the participants within a broad form Christianity which emphasises commitments to the centrality of the Bible, individual faith-commitments and conversions, a personal relationship with God through Jesus, and mission (Guest, 2008, p.1).

### Positionality

Researchers’ religious backgrounds and loyalties are important and controversial methodological concerns in Religious Studies, deserving explicit discussion and reflection, including self-reflection (Wilson, 2015, pp. 106–107). As an evangelical Christian, with no experience working in healthcare, I was complexly both ‘insider’ and ‘outsider’ to my participants. My religious background was known to participants, and when asked about my faith I was open about it. This shared religious identity afforded a shared discourse: I could communicate with participants freely, neither party concerned about using Biblical references, theological concepts, or terms such as ‘home group’ and ‘quiet time.’ Our shared faith also facilitated rapport and trust. Coar and Sim (2006) suggest that insider-interviewers ‘can gain potentially rich insights by capitalising on a shared culture and a common stock of technical knowledge, as well as feelings of collegial trust’ (p. 255). Certainly the participants’ language and discourse indicated that they perceived me to have a ‘common stock’ of religious knowledge, and a ‘shared’ religious culture, creating beneficial mutual religious empathy and understanding. Equally, conscious of methodological concerns regarding being ‘too close’ to or over-familiar with one’s subject matter (Connolly, 1999, p. 2), I drew upon experience researching Christianity both theoretically and empirically, and thus of balancing my own faith commitments against analytical rigour. Additionally, because I was learning for the first time how evangelicalism translated into healthcare work, I was at less risk of over-familiarity—for example, while a vocation discourse was familiar, I came to question its precise manifestations because of the unfamiliar medical setting. Moreover, the breadth of evangelicalism captured within the sample meant that I was not simplistically a religious ‘insider,’ but often found myself at some distance from particular ‘conservative’ or ‘liberal’ perspectives. This also required me to consistently reflexively evaluate my religious positionality. In particular I achieved this through keeping reflexive notes during data-gathering, analysis and writing, and discussing my findings and interpretations with academic peers (whose perspectives were, helpfully, different to mine, and who were able to highlight areas where my background had shaped my presentations). These practices together facilitated extensive reflection and a measure of triangulation.

### Analysis

As I received each set of reflections, and transcribed each interview, I refined a list of emerging topical codes, returning iteratively to previous data sources to ensure the whole dataset was eventually coded using the same full list, facilitated by qualitative data analysis software. These topical codes were organized into categories (e.g. ‘areas of medicine’; ‘ways of relating to God’) creating a nested ‘tree structure’ (top-level categories were not, themselves, used to code the data). During this iterative process, I noted observations and hypotheses which might inform subsequent purposive analysis.

Subsequent purposive analysis then took several forms: coding the whole dataset using a pre-existing list of emotions, in order to facilitate investigation of how emotions intersected with topics and themes; coding the whole dataset for sections particularly relevant to the research questions; critical close reading of coding reports (e.g. generating a report of everything coded at ‘vocation/calling’); in some cases, generating sub-codes in order to more closely interrogate a topic (e.g. data coded at ‘challenges’ was then coded again to tease out examples of ‘challenges specific to Christian medics’ as opposed to ‘challenges for all medics’); and running queries and using coding reports to examine the intersections of codes (for example, to show where in the dataset both ‘vocation/calling’ and ‘joy’ arose).

For the present paper, the following analytical points are notable: first, that it became evident early in inductive analysis and from participants’ easy movement between them that the terms ‘vocation’ and ‘calling’ overlapped closely, and so should be coded together rather than separately (the code was labelled ‘vocation/calling’); second, that the code I eventually labelled ‘vocation/calling’ was, in early stages of analysis, three separate codes: ‘vocation/calling,’ ‘God-given gifts,’ and ‘doing God’s work’. These were amalgamated because I found myself so often having to use them simultaneously, and I made a note to consider at a later stage how the three phenomena related; third, that the hypotheses that the medics’ discourse of calling operated on three ‘levels’ and related to various origins (see below) emerged during a critical close-reading of the ‘vocation/calling’ coding report and was subsequently tested (without further sub-coding); and fourthly, having noted a surprising range of emotional tones upon first reading the ‘vocation/calling’ coding report, I used the emotion codes to examine in detail different emotions the participants described in relation to or associated with calling.

## Data Analysis

### Identifying Three Levels of Calling

**Fig. 1:**
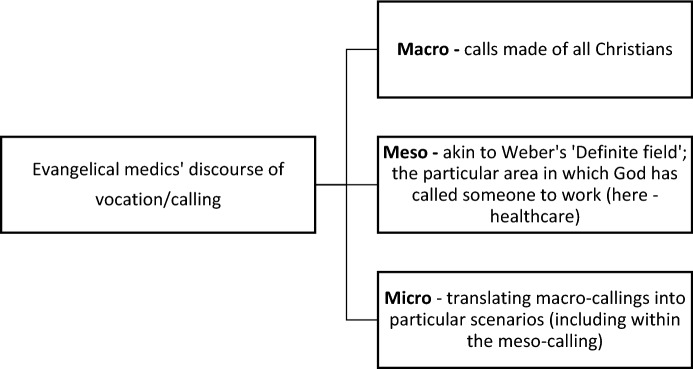
Visualizing three levels of ‘calling’

The participants’ discourse of ‘calling’ and ‘vocation’ operated on three levels. At the grandest level, they referred to ‘calls’ made of all Christians, often directly referencing biblical commands. In turn, participants considered how these translated into particular scenarios. Between the grand (macro)- and particular (micro)-levels was their medical work: a ‘meso-calling’ akin to Weber’s ‘definite field,’ to which they believed God had called or led them. We might visualize this as per Fig. 1:

The participants firmly considered medicine an arena amenable to living out Christian macro-callings, particularly to: love; showing compassion; and imitating Christ’s healing work through modern scientific medicine (Haynes & Kelly, 2006, p. 197; Knight & Kim, 2011, pp. 112–113; Chatters, 2000, p. 345). Like Cadge’s chaplains, the participants saw themselves as ‘channel[s] for divine love’ translating God’s call to love into specific scenarios (2013, p. 187). Junior doctor Martha described medicine as ‘in keeping with the life Jesus calls me to’ and her ‘platform [from] which I can easily interact with people who need…God’s love, and support, and care.’ Medical student Akua explained that Jesus:

taught everyone that we are called to care for each other...I definitely think that [medicine is] in line with how God wants us to treat each other…Jesus never told us why pain was there, but he did come and relieve people of it [and] treat it…that’s what we’re here to do.

In line with Fowler’s work, several medics particularly associated vocation with being ‘urged into the world’ in ‘service’ (1991, p. 50). Geriatrician Philip linked service with the specific ‘position’ he believed God had given him in which to enact it, describing being a Christian medic as ‘an amazing opportunity to serve people.’ Martha felt that the call to ‘service’ ought to translate into working specifically in public healthcare, explaining, ‘I’ve questioned my very strongly negative attitude to private healthcare, but I do really feel that if we’re called to be servants, and serve people who need it…why would we be called to serve in private medicine?’ Thus, this macro-calling shaped not only her meso-calling, but specific arena in which she sought to exercise her medical vocation.

Lewis, GP and former prison doctor, translated Christian ‘commands’ into the specific micro-settings of his work, in seeking to be ‘fully up-to-date […] fully attentive […] wise in my clinical judgements’:

I am commanded to love my neighbour as myself, to go the extra mile, to carry my cross. So I need to be the best doctor I can be—fully up-to-date (if only), fully attentive to the patient, her carers and family. I need to be wise in my clinical judgements and careful to do no harm.

For Akua, and others, the call to be wise stewards of God’s provision meant careful use of medical resources and ‘feeling less comfortable with having to open a second packet of something.’ For Philip, trying to ‘be as much like Jesus in my work as I can’ meant specifically showing compassion to both staff and patients. For Ginny this meant contravening the ‘ethos in hospitals’ used to pass ‘awful jobs to those at the bottom of the food chain.’ She also described her underlying ‘Christian ethos’ of:

valuing the dignity of those with mental health problems, dementia, learning disability, physical disability—and others rejected by society. For me, that leads to compassion for all, and behaviours which show value to those who are often marginalized [: for example] including someone with learning disability in a discussion.

Goodluck, a trainee cardiologist, described performing God’s ‘miracles’ by healing people through medical interventions. This was something Goodluck believed God had ‘put [him] in this place’ to do, linking macro-callings with his meso-calling:

One of the joys of my job is that I am performing God’s miracles every day. And my—that’s why I enjoy my job, I’m following my calling, I’m doing God’s work…[That] will look like arrogance. I am saying I am a miracle worker. [But] on a personal level, I know that God has supported me through school, medical school, and my jobs. I’m not here on my own. So it’s not that I’m arrogant, it’s that God has put me in this place as part of his work—and how humble that makes me feel cannot be underestimated.

Though he uses the term ‘miracles,’ Goodluck and the other participants did not have ‘miraculous’ healing in mind per se. Rather, most believed that, while God might and could heal through miracles, they had seen little evidence of this, and instead saw God at work through modern, scientific medicine.

### Knowing One’s (Meso-) Calling

Goodluck linked the particular ‘place’ in which God had ‘put’ him with the macro-calling to do ‘God’s work.’ He described the moment he identified medicine as his meso-calling, while applying to university:

I felt very challenged, I was about to make the largest decision of my life to date, and at no point had I consulted God in this process…so we prayed about it...And the spirit of God listens to what you’re saying even when you’re not praying, in that as soon as hands were laid on me and I closed my eyes, I had a very, very clear picture of—in the darkness, a stake being placed in the ground and two snakes wrapping themselves round it. And that is one of maybe two images I’ve ever had in my life…real, religious experience. And so after ten seconds I told them to stop praying, and I knew that this is—this is my life. And I’ve got to say, ever since, actually, that I’m doing what God wants me to do.

The ‘clear picture’ was, in Goodluck’s view, a religious experience—a direct, personal communication from God providing insight into his personal circumstances. Ginny, a GP-turned palliative care specialist, also linked her meso-calling with religious experience. She had felt God ‘speaking clearly’ at a time of career transition: ‘[I] sat in the churchyard to pray and looked up—the inscription [on] the gravestone was “peace after pain.” [I] felt this was God speaking to me re picking up palliative medicine again.’

Martha and child psychiatrist Simon both also had strong senses of vocation, but not as the result of religious experiences (what Martha termed ‘prophetic’ experience). Simon had ‘no doubt at all in my mind that [medicine] was a vocation’ and ‘what God wanted me…to do.’ He had felt this throughout his career, but believed it ‘even more so’ with retrospect, inferring from alternative ‘paths’ which were ‘redirected.’ Explaining her motives for pursuing medicine, Martha divorced her sense of vocation from ‘any kind of, prophetic thing,’ but nevertheless ‘definitely felt it was vocational’ and ‘felt really deeply that that was where everything was pointing…I felt like the qualities and characteristics God gave me were very well fitted for medicine.’ Indeed, she measured her career aspirations against these qualities:

There are [areas] where I don’t think I would be using the gifts God has given me… I wouldn’t want to do something that is hands off, like radiology, [and] I wouldn’t want to be a surgeon—it’s not what I’m gifted at.

Several participants were cautious about the terms vocation and calling, but shared beliefs that God had purposefully directed them to medical work. Junior doctor Jeremy felt having ‘a personal calling [was] overplayed in the modern Christian scene,’ but nevertheless, like Martha, had ‘tried to […] look at the person that I think that God has made me, and find a career that best suited those attributes. And that’s why I picked medicine.’ David, a GP with 35 years’ experience, tried not to ‘ponder’ vocation ‘too much, because I think you can get a bit big-headed.’ He nevertheless described:

[H]oping that by my faith, using prayer…that it will become obvious that I’m not in the right place. Umm. And I don’t seem to have had that message over the years…whether you’ve got a faith or not, you sometimes look at a crossroads and think that was a mighty strange coincidence, that all that just fell into place neatly…and if you have got a faith you might say well, there was maybe something there just nudging me in the right direction.

GP Elizabeth reverse-engineered a sense of assurance in God’s purpose for her life while at university. She explained that she never ‘felt particularly called to medicine…not that I consciously thought about,’ but nevertheless felt ‘assured that I was called [from] the fact that I was succeeding…and enjoying it…actually I was in the right place, and God had brought me to that place.’ Similarly, while junior doctor Hannah ‘never felt […] a clear calling’ she felt reassured during university by her belief that ‘God’s put me here…this is probably where I…should be.’ Her sense of calling had since ‘gradually’ grown in significance, and she had come to ‘see, on a daily basis, that I think this is where God wants me.’ Her sense of calling provided reassurance: ‘[When] I continue to see friends and colleagues quit…where that’s the case, and where you have a horrible rota, and consistent horrible days…because I have the belief that it’s my calling [I’m] more reassured.’ Her calling provided meaning and strength, an ‘interpretive lens’ to re-frame ‘horrible days’ and ‘nightmare scenarios or cases, where it feels like you want to give up.’

### Calling’s Positive Implications

Hannah and Elizabeth were not alone in drawing upon their senses of calling for resilience and perseverance. Psychiatrist Ruth felt her ‘sense of service to God’ bolstered her resilience, helping her to reach out to troubled patients ‘without being completely unhinged emotionally.’ Similarly, Martha described her colleagues ‘questioning “Why am I doing this, what’s the point, it’s so hard”.’ However, she explained ‘I just don’t feel that way—I feel like this is what I’m called to do…I feel a lot of purpose, and I think purpose is really good for, kind of, sustenance, perseverance, morale.’ In turn, she could ‘zoom out and [see] that was a really tough shift, and I need to work on how I better manage my stress [in] those situations, but I can still…come out of it, and see something positive.’

John’s sense of assurance in God’s plan for his career had helped him overcome obstacles:

God had a plan for me…and I had to take some drastic measures, like going [to] a country where [I didn’t] know the language, people, food, weather, culture and medicine! Everything was…against me…But I felt that that was God’s plan for me.

Of his decision to pursue surgery, John could with retrospect, ‘see now why that initial desire was there,’ and trace how everything had ‘fallen into place.’ He expanded, ‘you can’t just, sort of say it has happened by chance—[it was] God…using me for his purpose.’ Akua similarly described God ‘open[ing] doors, and clos[ing] different ones, at the right time, and I very much feel like I’m where I’m supposed to be.’ Akua and John’s words echo Harrison and Innes’ suggestion that a sense of vocation, even in secular contexts, often involves belief that ‘special factors’ have ‘intervened’ (Harrison & Innes, 1997, 18).

In addition to drawing resilience, determination and perseverance, many participants also felt bolstered by the ‘joy’ and ‘encouragement’ they derived from their callings. Simon framed medical work as ‘partnering with God’ in ‘meeting human needs.’ He found this ‘joyful.’ We saw above that Goodluck’s sense of fulfilling his calling made him feel ‘humble’ and brought him joy. Martha explained that working in medicine had ‘encouraged me in my faith as when I work, I feel that I am fulfilling my calling. I feel God equipped me to do medicine & do it well, so I feel encouraged & blessed to live this out.’ Jeremy found it ‘joyful to be able to spend so much time exercising the gifts that God has given me.’

Several participants also used calling as a lens on the future. While Martha felt drawn to cardiology, she contentedly recognized that ‘God [c]ould want me to choose a different career path…I’m not really concerned about it, because I feel that…God will show me where he wants me to be.’ David, at the other end of his career, pondered: ‘If you do feel that this has been a God-led career…what do you do now?…isn’t it job done?…But of course, if you’ve got a good strong faith, you just say, “I’m ready and waiting”.’ Like the students in Williams’ study, having seen God’s facilitation of his purposes for their lives thus far, these doctors felt confident of future direction (Williams, 2013, pp. 264–267).

### A ‘stick with which to beat you,’ or an ‘Idol’? Negative and Ambiguous Implications

Such positive consequences were important elements of the participants’ understandings and experiences of calling and vocation. In addition, however, the participants also presented more ambiguous, and at times negative, conceptualizations. Lewis described medicine as his vocation on several occasions, but emphasized its dual potential to both bolster resilience and add burdens. He wrote, ‘a sense of calling or vocation may help [Christian doctors] through the hard times. But it can also be a stick to beat you with (by others or yourself).’ He painted this hypothetical picture:

[Say] you say you’re “called,” and I’m your boss, so I say “Great, she says she’s called, you know—so she can do every Friday night on call—she can do the extras, she can see the difficult patients […] because that’s her—her calling.”

Recognizing the challenges of achieving work-life balance and treating ‘difficult’ patients, Lewis suggested a doctor’s calling might become a stick with which others could beat them. Equally, he suggested it was perhaps likelier that doctors with a sense of calling might use it as a stick with which to beat themselves. He explained, ‘you can beat yourself up about it as well. If I’m called to this people, then I must spend every possible hour, minute, day serving them.’ This potentially harmful self-inflicted pressure to fulfil a calling was also latent in Philip’s reflections:

I’ve got to live [my calling] out as well as possible. You know, making the most of every opportunity that I’m given by God to serve him in the area that I’m given to serve…I wouldn’t be able to sustain my work ethic, of working really, really hard, without that Christian basis. Certainly I wouldn’t be…starting early and staying late unless I cared deeply.

Philip’s desire to serve ‘as well as possible,’ making ‘the most of every opportunity,’ drove him in a potentially burdensome way (and it was unfortunate that I could not conduct Philip’s follow-up interview—I had hoped to explore this further).

While Martha’s sense of vocation bolstered her morale, she also struggled with guilt, writing:

When colleagues talk badly and unsympathetically about each other behind each other's backs—I am conscious of the different attitude we have as Christians re: forgiveness, turning the other cheek, looking compassionately on others, showing grace. And—when I am tempted to join in, or do join in, I feel guilty as I think of the grace Jesus calls us to.

Martha acknowledged calls to behave distinctively, with compassion and grace, and by ‘turning the other cheek,’ and felt guilty where she did not enact these. She made similar points about patience and compassion. Falling short of these macro-callings burdened Martha with guilt. Retired psychiatrist Sarah upheld a biblical ideal of ‘loving all patients.’ However, she felt this ‘unfortunately [got] eroded by events’ and over time. She found herself asking ‘Why am I bothering with this? Because it’s not going to change anything.’ Hannah felt the culture of cynicism and disillusionment cultivated by some of her NHS colleagues inhibited her ability to show love, and thus, at times, she doubted her ability to ‘live out’ her faith as a doctor. Medical work often facilitated the translation of macro-callings into micro-contexts: when it did not, this could result in significant negative feelings of guilt, doubt and worry.

More ambiguously, both Peter and fellow student James described holding lightly to medicine as their meso-calling, not least because they believed that God might call them another. Here, Weber’s ‘definite field’ ought to be pluralized. James cited the biblical precedent for ‘re-calling’:

I might just one day have to drop out of medicine. Because [I’m] called to something else, you know! There’s so many people in the Bible that did something else…Don’t get comfortable, you know—Jesus was a carpenter, David was tending his sheep…and I think it’s the same. And you just have to be attentive.

Peter, similarly explained he didn’t:

want to get to a point where I didn’t feel comfortable with the idea of leaving the medical profession if God wants…what God calls me to, I want to be able to do…I want to be open to the fact that I may get to 30 and may think God’s saying “I want you to drop medicine.”

Peter recognized that medicine might only be God’s current plan for his life. Citing Psalm 119, he reflected that God is a ‘lamp to our feet [but] doesn’t illuminate a nice map throughout all of our life…sometimes he shows us the next step only when he wants to.’ Fowler’s work is helpful here, for he portrays vocation as process, rather than as static, describing ‘ongoing discernment of one’s gifts [and the] means and settings’ in which they are to be used (Fowler, 1991, pp. 50, 118–121). This allows for vocations to change and shift, important dimensions for understanding Peter and James’ concerns.

Peter was also keen to avoid reaching ‘a point where medicine is too much my life and too much my idol…And I suppose that comes back to identity—people being committed only to medicine.’ Employing a theological narrative of idolatry, Peter recognized that medicine might become important to his faith’s detriment, inhibiting his ability to follow God’s callings elsewhere. Similarly, James hoped to always be ‘a Christian first and a doctor second’ lest medicine become all-consuming at his faith’s expense.

Equally, though, several participants gratefully found that their faith afforded relief from pressures to chase medical success. Peter described his senior colleagues for whom ‘being a surgeon means everything,’ before ‘contrasting that with knowing that my hope is in Christ, and knowing that I don’t have to go there and make my job the best thing and the most important thing.’ Similarly, Jeremy was ‘very glad to not have to strive to be anyone/anything; as [being a doctor] isn’t the most important thing in my life.’ He contrasted this with those whose ‘sense of being a doctor is a massive part of their identity [and] self-worth. If you’re a Christian, that works the opposite way…your sense-of-self-worth is…defined by God’s attitude towards you…so there’s less pressure.’ Thus, even as participants felt they must hold lightly to their medical callings, lest they become idols, or too much of their identity, potentially inhibiting them from following God’s lead elsewhere, they were also relieved that they could hold these lightly amid pressure to pursue medical excellence and ambition above all else. This interaction between the evangelical logic of vocation and beliefs relating to idolatry and identity therefore had ambiguous consequences: participants were relieved from burdensome ambition, but risked conflicted priorities in relation to their meso-calling, insofar as medicine could sustain many Christian macro-callings, yet might also contradict calls to prioritize their faith and avoid idolatry.

## Discussion

### Nuances and Complexities in Narratives of Calling

**Fig. 2:**
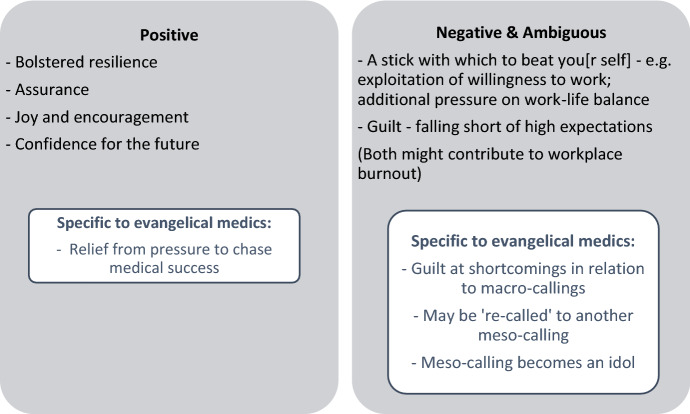
Mixed implications of a sense of calling

While their language varied, a sense of vocation was consistently significant for the participants, whether they attributed this to clear communication from God, ‘felt deeply’ that medicine was right for them, saw a medical career as the outworking of God-given skills, or had reverse-engineered their sense of meso-calling to medicine from their success in the field. In order to understand these, we must appreciate several nuances: first, distinctions between three different levels—macro, meso and micro—on which calling operates (Fig. 1); second, that senses of one’s meso-calling can have myriad sources, and might or might not intersect with evangelical narratives of God-given gifts or religious or ‘prophetic’ experiences. Bosk’s observation that vocation may not, as Weber implies, relate to an inner voice inspiring determined passion, is here translated into the context of evangelical Christianity (Bosk, 2014, pp.376–377); and thirdly, that while a sense of vocation can have positive consequences, it can also have negative and more ambiguous consequences (Fig. 2). In addition to showing that vague, imprecise use of the term vocation is insufficient to appreciate evangelical medics’ understandings, uses and implications of such discourse, this paper thus highlights the multiple sources and three-levelled discourse identified here offer greater precision for understanding evangelicals’ conceptions of vocation than has been offered before. Scholars should attend to these and other nuances easily masked by imprecise usage of the terms calling and vocation, and further consider how these intersect with other religious narratives for evangelicals.

### Mixed Implications: Returning to Weberian Analysis

The evangelical medics echoed Williams’ presentation of calling as an ‘interpretive lens,’ grounded in belief that God is active in the world, leading believers to see themselves and their work as part of God’s ‘plan’ (Williams, 2013). Believing God had called them to work in the ‘definite field’ of medicine, the evangelical medics saw their everyday, secular work as ‘religiously significant’ (Weber, 2013; Williams, 2013). There was further overlap between these scholars’ conceptualizations and the medics’ presentations of the positive implications of calling. Several participants benefitted from a sense of ‘meaning’ and ‘purpose’ in the name of ‘serving’ God’s ‘cause’, enacting Christian macro-callings and utilizing God-given gifts (Weber, 2004, p. 40; Fowler, 1991, p. 120). This both brought joy and provided resilience, encouragement, assurance and determination, echoing Williams’ assertion that evangelicals derive ‘strength, support and guidance’ from their callings (Williams, 2013, p. 257). It is important to recognize that a sense of vocation can valuably bolster evangelical medics in these ways, representing a positive emotional and interpretive resource when they faced obstacles or discouragement.

However, several medics were also equally clear about the more negative and ambiguous ramifications of vocation and calling. For example, several medics felt that they must hold lightly to their sense of medical meso-calling, since God might well call them to work in a different field. This theological narrative of ‘recalling’ interacted complexly with Peter and James’ beliefs that pursuing medicine was part of God’s plan for their lives. This introduced theological tension into their understandings of medical vocation. The same could be said regarding the theological framework of idolatry: while several participants understood their vocation to mean God had called them to be a doctor, this vocational identity needed to be held in balance with the risk that it might become all-consuming. Here, Weber’s caution about callings which ‘diminish all else’ is embedded within evangelical logic: for Peter and James the risk was that the meso-call might diminish its own religious significance, instead becoming an idol (Weber, 2004, p. 7, 10). Williams and Fowlers’ examinations of Christian vocation identify no such theological ambiguities, which were important considerations for these evangelicals as they considered their work. By contrast, Weber’s work encourages us to view vocation with a degree of suspicion.

Several participants also highlighted ways in which vocation could—consciously or otherwise—become a stick with which they or others might beat them, reminiscent of Weber’s suggestion that *Beruf* can facilitate exploitation (Weber, 2013, p. 106). Harrison and Innes note a similar risk, suggesting their commitment and willingness to be self-sacrificial in a vocation can lead to junior staff in particular being placed under ‘unfair pressure’ by senior staff (Harrison & Innes, 1997, pp. 11–12). They also suggest doctors may also put unfair pressure upon themselves, which could harm both themselves and their patients (Harrison & Innes, 1997, pp. 11–12, 23). Lewis recognized this risk, illustrating some doctors’ desires to ‘spend every possible hour, minute, day, serving’—a latent risk detected in Philip’s reflections. Lewis thus pointedly noted the importance of enacting Christian callings beyond medical work, as well as within it. This also evokes Weber’s presentation of *Beruf* as a double-edged sword, edifying yet burdensomely exclusive and demanding, potentially ‘diminishing all else in life’—what Harrison and Innes, citing Volf, term ‘divinization.’ Relatedly, the above has also showed that evangelical medics may simultaneously find that their sense of vocation bolsters them, but creates high self-expectations, based on macro-callings (to, for example, distinctively compassionate and gracious behaviour) of which they deem themselves to fall short. This resulted in burdensome feelings of guilt. Such a cycle of expectation and guilt might easily lend itself to burnout and over-work.

On the basis that it identifies such negative and ambiguous implications of vocation alongside its more positive manifestations (see Fig. 2) this paper advocates continuing in the direction identified by Weber, applying a hermeneutic of suspicion to vocation as it is used by evangelicals and manifests in their lives. Its relationship to other theological narratives—such as idolatry, or re-calling—can be complex. Macro-callings to, for example, service or acting with distinctive compassion and grace, can also create burdensome consequences for evangelical individuals, suggesting that recognizing the three-tiered logic of calling aids understanding of vocation’s complexity and mixed implications. Having an eye to these nuances will encourage and elicit richer understanding of its implications. This is not only important with respect to medicine, but potentially for all jobs which confer a significant sense of identity upon evangelicals. In addition to encouraging richer academic understanding of vocation, this observation will enable those who seek to support evangelical Christians as they aspire to enact their macro-callings in diverse meso-spheres and micro-scenarios to do so with awareness of the burdens vocation can create, as well as its positive implications. Future research could usefully explore whether and how raising awareness of vocation’s mixed implications might facilitate evangelicals’ self-understanding and awareness, with a view to finding ways of using detailed understandings of calling to break or prevent cycles of expectation, guilt, burnout and over-work.

### Vocation and Workplace Burnout

Over-work, burnout and exploitation are, moreover, widespread concerns in contemporary Western medicine, independent of medics’ religious backgrounds. The literature review highlights different ways in which a discourse of vocation can interact with these concerns. Traynor identifies ways in which a discourse of vocation might paper-over exploitation of nurses’ willingness to go the extra mile and ‘put [themselves] out’ and allow work to ‘encroach’ on their personal time (1999). As Harrison and Innes identified in the late 1990s, recognizing the risk that over-emphasizing vocation might pose to medics is an important step in responding to ways in which work and life can become imbalanced, to the detriment of both patients and practitioners. Contrastingly, when Astrow (2013) identifies vocation as a potential counterbalance to burnout, he does not recognize that a sense of vocation might also, ultimately, fuel burnout. As Fig. 2 reinforces, a number of the concerns evident in the above narratives regarding guilt, and exploitation of one’s own or another’s deep-seated investment in their vocation, are not concerns unique to evangelical medics—particularly since, as we have seen, a discourse of ‘vocation’ is often used without any specific religious entailments. As such, this paper affirms those scholars who question uncritically positive and neutral uses of ‘vocation’ in medical contexts more broadly. Future research might therefore not only explore the consciousness-raising effects of exploring vocation’s mixed implications among evangelicals, but also among healthcare practitioners of diverse religious and non-religious backgrounds. By taking a leaf from Weber’s interrogation of *Beruf*, scholars and healthcare trainers and mentors might all be better attuned to its complex manifestations in, and interactions with, practitioners’ identities and experiences, and better placed to support those practitioners.

## Study Limitations

The present study was completed with a relatively small sample of evangelical medics in one national context (England). Focussing on this national, religious and occupational context limits the study’s generalizability, though did facilitate in-depth qualitative exploration. Within this context, while care was taken to make sampling and recruitment inclusive, the sample is not statistically representative. Mixed-methods or quantitative testing might establish the extent to which the views expressed in this study resonate within representative samples of English evangelical healthcare professionals. Further qualitative, mixed-methods and quantitative research would also be needed to explore whether the findings presented here resonate with: evangelicals working in other fields; medics of other (or no) religious backgrounds; and evangelical medics working in other national contexts.

## Conclusion

This paper has explored conceptions of calling and vocation held by evangelical medics, and the implications of these for their work and identities. Amid varied language, participants had a shared logic of calling, operating on three levels. Medical work was seen as a God-given purpose, but this meso-calling was also an arena for enacting macro-callings made of all Christians. The participants translated these macro-callings into micro-scenarios within their meso-calling. Many derived reassurance, resilience and joy from their senses of calling, and their belief that they were fulfilling these. Some, however, also recognized darker and more ambiguous ramifications. Thus, while it found significant support for Williams’ presentation of calling among evangelicals, and Fowler’s broader Christian understanding, this paper has also urged caution: conceptualizations of calling ought not to lose sight of their potentially negative consequences. Rather, a cautious eye ought to be extended over evangelical constructions of calling, recognizing their potential to over-burden, diminish other areas of life, or induce taxing guilt, as well as their ambiguous relationship with narratives of idolatry and re-calling. These conclusions regarding the importance of recognizing in vocation both the potential to bolster and the potential to exploit, moreover, echo broader concerns regarding discourse of medical vocation. While it is right to identify and comment upon the positive consequences a sense of vocation might have, this must not be the point at which critical examinations of vocation end. We reach wistfully for the restoration of historical discourses of ‘medical vocation’ at our peril if we do so uncritically.

## Data Availability

Not applicable.

## Availability of Data and Material

To be confirmed.
